# Cortisol Levels Chorelated with Exposure to Alcohol Related Visual Stimuli in Patients with Alcohol Use Disorder

**DOI:** 10.1192/j.eurpsy.2024.863

**Published:** 2024-08-27

**Authors:** A. Mihai, M. Valeriu

**Affiliations:** ^1^Psychiatry, University of Medicine, Pharmacy, Technology and Science; ^2^Mureș County Hospital, Targu-Mures, Romania

## Abstract

**Introduction:**

The mechanism of craving is not yet fully understood. It implies numerous factors contributing to the decisions an individual has to ponder when faced with a stimulus that has resemblance with the previous experiences related to it. Neural pathways implying the reward mechanism play a significant role in the interpretation of visual, auditory, olfactive stimuli, polarizing the perception towards positive or negative experiences with that substance of abuse.

**Objectives:**

In this study we focus on the cravings related to alcohol use, in a sample of patients admitted in hospital due to alcohol use disorder pathologies, providing the fact that Romania has the 2nd highest prevalence of heavy episodic drinking at least once a month (35% of adults, in a statistic published by Eurostat in 2019).

**Methods:**

We included 30 patients with alcohol use disorder. The PACS (Penn Alcohol Craving Scale) was used to assess the severity of craving in the week prior to the hospital admission.Before visualising any alcohol related cues using VRET, patients will have a half hour of group therapy to lower levels of anxiety. Cortisol and blood sugar will be measured after this half hour to set a baseline . Afterwards, using VRET, subjects will be asked to watch a number of visual stimuli that will include cues to alcohol consumption and different types of beverages. Half hour after visualising cues of alcohol, the craving will be assessed by measuring blood sugar and salivary cortisol levels once again. Completing these measurement, patients will be asked to complete the PACS scale one more time to corelate the patients craving with the biological findings. Blood sugar levels will be measured with a blood glucose meter with test strips. Cortisol levels will be measured using salivary levels of cortisol. We choose measuring the salivary levels of cortisol, due to the fact that using this method, the biological active, free cortisol. Measurements of the serum cortisol indicate the total quantity, but not the biologically effective cortisol.

**Results:**

Visual stimuli of alcohol, with the help of VRET modifies the autonomous glucocorticoid secretion, and provide objective information complimentary to the each individual’s craving assessment

**Conclusions:**

There are a great number of strong ties between alcoholic craving in patients and endogenous shifts in cortisol secretion. We aimed towards a better understanding on craving in patients hospitalised for AUD. Other directions for future research are to find out if it possible to consider craving a form of stress or if we could limit craving, by limiting stress.

**Disclosure of Interest:**

None Declared

